# Dietary Change Scenarios and Implications for Environmental, Nutrition, Human Health and Economic Dimensions of Food Sustainability

**DOI:** 10.3390/nu11040856

**Published:** 2019-04-16

**Authors:** Canxi Chen, Abhishek Chaudhary, Alexander Mathys

**Affiliations:** 1 Laboratory of Sustainable Food Processing, ETH Zurich, Schmelzbergstrasse 9, 8092 Zurich, Switzerland; canxi.chen@hest.ethz.ch (C.C.); alexander.mathys@hest.ethz.ch (A.M.); 2 Department of Civil Engineering, Indian Institute of Technology (IIT) Kanpur, Kanpur 208016, India

**Keywords:** sustainability, dietary changes, health impact, nutrition quality, environmental impacts

## Abstract

Demand side interventions, such as dietary change, can significantly contribute towards the achievement of 2030 national sustainable development goals. However, most previous studies analysing the consequences of dietary change focus on a single dimension of sustainability (e.g., environment) using a limited number of indicators and dietary scenarios. A multi-dimension and multi-indicator analysis can identify the potential trade-offs. Here, starting from the current food consumption data (year 2011), we first designed nine alternative dietary scenarios (healthy Swiss diet, healthy global diet, vegetarian, vegan, pescatarian, flexitarian, protein-oriented and meat-oriented diets and a food greenhouse gas tax diet). Next we calculated three nutritional quality (nutrient balance score, disqualifying nutrient score, percent population with adequate nutrition), five environmental (greenhouse gas, water, land, nitrogen and phosphorus use), one economic (daily food expenditure) and one human health indicator (DALYs) for current and alternative diets. We found that transition towards a healthy diet following the guidelines of Swiss society of nutrition is the most sustainable option and is projected to result in 36% lesser environmental footprint, 33% lesser expenditure and 2.67% lower adverse health outcome (DALYs) compared with the current diet. On the other extreme, transition towards a meat or protein oriented diet can lead to large increases in diet related adverse health outcomes, environmental footprint, daily food expenditure and a reduction in intakes of essential nutrients (for Vitamin C, Fibre, Potassium and Calcium). We found that shifting to the vegetarian and vegan diet scenarios might lead to a reduction in intakes of certain micronutrients currently supplied primarily by animal-sourced foods (Vitamin B_12_, Choline and Calcium). Results show that achieving a sustainable diet would entail a high reduction in the intake of meat and vegetable oils and a moderate reduction in cereals, roots and fish products and at the same time increased intake of legumes, nuts, seeds, fruits and vegetables. We identify several current data and research gaps that need to be filled in order to get more accurate results. Overall, our analysis underscores the need to consider multiple indicators while assessing the dietary sustainability and provides a template to conduct such studies in other countries and settings. Future efforts should focus on assessing the potential of different interventions and policies that can help transition the population from current to sustainable dietary patterns.

## 1. Introduction

The food system is highly related to the Sustainable Development Goals (SDGs) involving hunger, nutrition and health, climate change, natural resources, biodiversity and socioeconomics [1,2]. Agriculture is the largest consumer of fresh water and the second largest contributor to greenhouse gas emissions worldwide [3,4]. Food consumption accounts for 20–30% of the environmental burden of total consumption in Europe [5]. At the same time, non-communicable diseases (NCD) or chronic diseases, accounted for 71% of all deaths globally in 2015 [6], while dietary risks (e.g., high consumption of red meat) and metabolic risks (e.g., high body-mass index) are predominantly associated with chronic disease burden. Diet plays a central role in the health of the population. There is an urgent need to limit environmental damage in order not to transgress the planetary boundaries [7] while providing a nutritious diet to a growing world population.

Previous studies have found that demand side interventions such as dietary shift (e.g., towards vegan, vegetarian, Mediterranean diets) can have positive consequences for both human health and climate change [8,9,10,11,12,13]. However, others have found that shifting from current to vegetarian, vegan or even flexitarian diets carries the risk for deficiency of certain micronutrients that are currently supplied primarily through animal-based products [14,15]. In general, shifting to plant-based diets not only results in greenhouse gas emissions reduction but also other environmental benefits such as water savings [16,17,18,19].

Recent evidence shows that different dietary scenarios can have widely varying consequences on different dimensions of sustainability [20]. It is therefore important to explore multiple scenarios of dietary change employing multiple indicators of sustainability in order to identify potential trade-offs and get a holistic and comprehensive picture of the consequences of dietary change.

Studies calculating more than one indicator of dietary sustainability are emerging [14,21,22,23,24,25,26]. However, they are either restricted to a particular environmental aspect (e.g., greenhouse gas (GHG) emissions), a limited number of nutrient (e.g., calorie, protein) or health aspect while not taking into account intake of micronutrients, health effects of multiple food groups (e.g., pulses) or ignoring certain health risk-outcomes (e.g., obesity).

Recent years have seen development of new approaches to quantify the nutritional quality scores of individual diets taking into account the intake of essential and harmful nutrients and comparing it to their recommended levels [14,27,28]. Assessment of human health consequences due to a shift in food consumption behaviours is also increasingly becoming common due to availability of food group-specific risk factors from long-term epidemiological studies [29]. Regarding the environmental dimension, the emission factors and footprint values for different food items compiled through meta-analysis of life cycle assessment studies are now available enabling the calculation of damage across multiple environmental categories [20,30]. While dietary choices determine health and environmental outcomes, they in turn are driven by food cost especially in low-middle income countries [31]. Healthy and environmental-friendly diet from sustainable food systems should be made economically affordable for communities. A reduced household expenditure on daily food purchase can help and drive the dietary shift.

In terms of various dietary change scenarios often employed, vegetarian and vegan diets are the most common ones. While the vegan diet excludes all animal-sourced foods, the vegetarian diet can include dairy products and an ovo-lacto vegetarian diet can include both egg and dairy products. Another variant, the pescatarian diet, excludes meat but can include fish. Much more relaxed than above, a flexitarian or semi-vegetarian diet, is a predominantly plant-based diet with the occasional inclusion of meat or fish [32].

Few studies to date have integrated environment, nutrition, economic and human health indicators to assess the consequences of different dietary change scenarios. The aim of this study is to fill this research gap and demonstrate the calculation of multiple indicators of sustainability for a suite of dietary change scenarios through a case study of Switzerland and explore the potential synergies and trade-offs across different indicators.

## 2. Materials and Methods

### 2.1. Dietary Scenarios

The current (year 2011) Swiss consumption of 82 food items was obtained from the Food and Agriculture Organization of the United Nations (FAO) food balance sheet [33]. We call it our reference scenario (REF) in this analysis. Healthy global diets (HGD) were constructed according to a description provided in another study [9]. The implementation of global dietary guidelines [34] on healthy eating and that people consume just enough calories to maintain a healthy body weight are assumed.

In addition, we developed a food greenhouse gas tax (TAX) scenario that provides an alternative diet as a consequences of levying a greenhouse gas (GHG) tax on food products. The TAX diet was derived based on the approach presented by Springmann et al. [35] showing how the consumption amounts of different food items are affected by changes in their price based on their GHG emissions content. This was calculated by combining the data on current food consumption (from FAO), with their GHG characterization factors (e.g., gCO_2_eq per g of beef; from Springmann et al. [20]), and the price and expenditure elasticities (from Tiffin et al. [36]) along with a food greenhouse gas tax of 96 Swiss Franc per tCO_2_-eq (~96.36 US $) adopted from guidelines presented by the Swiss Federal Office for Environment. Note that the data on own-price and cross-price elasticities for food items [37] are not available for Switzerland yet and therefore we used the UK-based elasticity parameters which have also been used for other high income countries such as Australia [35]. We aggregated the Swiss food consumption and their price data to the commodity detail of the elasticity estimates.

The amount of 82 food items under the rest of the seven alternative dietary scenarios were calculated based on the ratio of their amounts in current and alternative diets available from Eggenberger et al. [38]. The seven alternative diets were as follows: vegan (VGN), lacto-ovo vegetarian (VGT), lacto-ovo pescatarian (PST), flexitarian (FXT), protein-oriented (PTO) and meat-oriented (MTO) diets, as well as an eating pattern following the recommendation of the Swiss Society for Nutrition (RSN). The flexitarian diet is much more relaxed than the above three and includes small amounts of animal-sourced products. Shifted in an opposite way, the latter two scenarios contain generous amount of eggs and dairy product (protein-oriented) or meat (meat-oriented) compared to the current diet. The Swiss Association of Vegetarians estimates that approximately 3% of the population are vegetarian based on surveys between 1997 and 2013. Rough estimates report that one-tenth of these vegetarians follow a vegan diet and the rest are lacto-ovo vegetarian eating pattern [39]. More than half (~54%) of the population claim that they eat meat 6 to 7 days a week [40].

Table 1 shows the average intake of different food groups per capita per day in Switzerland under current and alternative dietary scenarios.

### 2.2. Human Health Impact Assessment

We analysed the health outcomes associated with changes in food consumption by using a comparative risk assessment framework with four disease states and eight dietary and weight-related risk factors. Reduced Disability-Adjusted Life Years (DALYs) was the indicator for our human health impact analysis. DALY measure the quality-adjusted life years, which is by definition the sum of Years of Life Lost (YLL) and the Years Lived with Disability (YLD). Here we calculated YLL as the number of deaths multiplied by the standard life expectancy at the age at which death occurs for a given cause, region, sex and age.

In our analysis, the four selected disease states were ischemic or coronary heart disease (CHD), stroke, type 2 diabetes mellitus and total cancer. The six diet related risk factors were low consumption of fruits, vegetables, legumes, fish, nuts & seeds and high consumption on red meat. The two weight-related risk factors are being overweight (BMI ≥25 to <30 kg/m^2^) or obese (BMI ≥ 30 kg/m^2^). We did not consider underweight (BMI < 18·5 kg/m^2^) here because the dietary calorie intake among Swiss generally make them meet the lower range of normal BMI. Dietary and weight-related risk factors have a dose-response effect on diseases risk. For example, every 100 g fruit increase per day would reduce approximately 23% of stroke risk (mean RR = 0.77, 95% CI 0.70–0.84). The relation between the effect of risk factors and health outcomes were quantified by relative risk (RR) from various meta-analysis and systematic review of epidemiological studies (Supplementary Table S1) [29]. In line with the existing evidence, we included non-linear dose-response relationships for fruits, vegetables, fish, nuts and seeds and assumed linear dose-response relationships for the remaining risk factors [29]. For example, Aune and colleagues [41] reported that most of the reduction in disease risk for nuts was observed up to an intake of 15–20 g/day, so we should not cumulate further health benefit from the increased nuts intake once the intake level reaches 20 g/day.

We estimated the reduced YLL and DALYs attributed to dietary and weight-related risk factors by calculating population impact fractions (PIFs) and applying age-specific and disease-specific mortality rate. PIF was the proportion of disease case that would be avoided when the risk exposure was changed from the counterfactual situation to the baseline situation, while the distribution of other risk factors in the population remained unchanged. By definition, PIF was calculated as follows:

(1) $$PIF=\frac{\int RR\left(x\right)P\left(x\right)dx-\int RR\left(x\right)P^{\prime }\left(x\right)dx}{\int RR\left(x\right)P\left(x\right)dx}$$

where (*x*) is the relative risk of disease for risk factor level *x*; (*x*) is the number of people in the population with risk factor level *x* in the baseline scenario; $P^{\prime }\left(x\right)$ is the number of people in the population with risk factor level *x* in the counterfactual scenario.

Here we regarded dietary and weight-related risk factors as discrete:

(2) $$\mathrm{PIF}=\frac{\sum_{x}P_{x}\times RR_{x}-\sum_{x}P_{x}^{\prime }\times RR_{x}}{\sum_{x}P_{x}\times RR_{x}}=1-\frac{\sum_{x}P_{x}^{\prime }\times RR_{x}}{\sum_{x}P_{x}\times RR_{x}}$$

We first calculated reduced death number (DN) by multiplying PIF with death rate (DR) and the population size (N) of the considered population group (Equation (3)), while YLL was the product of DN and predicted life expectancy (E) [42] (Equation (4)). DALY was calculated by the ratios of deaths and the age and gender-specific mortality-DALY derived from World Health Organization (WHO) estimated for the year 2012 [43].

In our model, we estimated the health impacts (e.g., life years or DALYs per year) by shifting from reference (current) diet to alternative scenarios for the whole Swiss population. For example for YLL in Equation (5), the impact due to each disease was the sum of the outcomes in different subgroups, which were gender- and age-specific. In Equations (3)–(5), index *d, j, k, f and s* referred to the identity of disease, gender, age group, risk factor and diet scenario, respectively.

(3) $$DN_{d,j,k,s,f}=PIF_{d,s}\times DR_{d,j,k}\times N_{j,k}$$

(4) $$YLL_{d,j,k,s,f}=DN_{d,j,k,s,f}\times E_{j,k}$$

(5) $$\mathrm{total}\;\mathrm{YLL}=\sum_{d,j,k,s,f}YLL_{d,j,k,s,f}$$

Here we adopted age- and gender-specific population and disease-specific mortality data from United Nation Population Division and Global Burden of Disease estimates of the Institute for Health Metrics and Evaluation (IHME), respectively. In our model, we estimated the health impact by shifting from reference diet to alternative scenarios for the average Swiss population.

For dietary risk factors, under a particular dietary scenario (e.g., current diet, vegan diet, etc.), we assumed that the whole population of a region is subjected to the dietary risk associated with the regional consumption level $C_{f}$ measured in gram per capita per day and that risks began increasing at zero consumption were unbounded [44]. We adopted standard serving sizes ($c_{serv}$) of 100 g for all food groups. Taking into account the consumption levels $c_{s}$, the relative risk associated with the consumption-based risk factor *f* = {*red* *meat*, *fruits, vegetables, legumes, fish, nuts & seeds*} was calculated by raising the risk factor to the power of the consumption level over the serving size, $RR_{f,s}=R{R_{f}}^{c_{s}/c_{serv}}$ (see Springmann et al. [44]). The associated PIF for risk factor *f* and disease/cause of death *d* was expressed as:

(6) $${\mathrm{PIF}}_{d,f,s}^{diet}=1-\frac{R{R_{f,d}}^{c_{s}/c_{serv}}}{R{R_{f,d}}^{c_{ref}/c_{serv}}}=1-R{R_{f,d}}^{\frac{C_{s}-C_{ref}}{c_{serv}}}$$

where $c_{ref}$ denotes the consumption level in the baseline scenario, $c_{s}$ the consumption level in the counterfactual (alternative dietary) scenario.

We calculated the combined disease and mortality burden of changes in all six dietary risk factors by using equation:

(7) $$PIF_{d,s}=1-\underset{f}{\prod }(1-PIF_{d,f,s})$$

For the weight-related health impact assessment, we first estimated the prevalence (as % of total population) of overweight ($P_{overweight}$) and obesity ($P_{obesity}$) in Swiss population under different dietary scenarios. The prevalance estimation was by regression models (Equations (8) and (9)) establishing from pairing FAO food availability data for WHO data on the prevalence of overweight and obesity, respectively, for the year 1980–2009 globally [9,44].

(8) $$P_{overweight}=0.02462\cdot \mathrm{kcal}-29.67965$$

(9) $$P_{obesity}=0.01000\cdot \mathrm{kcal}-14.98936$$

In Equations (8) and (9), kcal is the total daily energy intake measured by kilocalorie per capita per day. The weight-related population impact fractions (PIFs) accounted for the weight status of different population fractions and the associated risks. Following Equation (2), they were expressed as:

(10) $${\mathrm{PIF}}_{d}^{weight}=1-\frac{\sum_{w}p_{w}^{scn}\cdot RR_{w,d}}{\sum_{w}p_{w}^{ref}\cdot RR_{w,d}}$$

where *w* = {*normal*, *overweigt*, *obese*} and the relative risks *RR* are differentiated by disease *d* and weight category *w*; and the population fractions *p* are differentiated by weight category; they are indexed by *ref* and *scn* for their levels in the baseline and counterfactual scenario, respectively. For uncertainty analysis, we accounted for the major uncertainties by using error propagation. With the confidence intervals (CIs) of the relative risk parameters, we calculated uncertainty intervals associated with health impact indicators. See Supplementary Table S1 for values of all parameters used in health modelling.

### 2.3. Nutrition Quality Analysis

We calculated three complementary indicators of the nutritional quality of a national average diet following the framework described in Gustafson et al. [45] and Chaudhary et al. [14]: nutrient balance score (NBS), disqualifying nutrient score(DNS) and percentage of population with adequate nutrition intake (PAN). The first indicator NBS reflects the micronutrient density of individual diets or food items [14,28,46] (i.e., essential nutrient amounts per unit calorie intake), while the PAN is a population level indicator and estimates the proportion of a total population that get adequate amounts of essential nutrients. The DNS indicates whether the intake of unhealthful nutrients is above maximum recommended levels or not [28].

We calculated the nutrient balance score (0 < NBS < 100) of national diets using the intake values of calories, all 23 essential nutrients and their reference daily intake (RDI) values (full details in Fern et al. [28]; Chaudhary et al. [14]). Since it is normalized by total calorie intake, the NBS reflects the micronutrient density of daily average diet. To this end, we first calculated the qualifying index ($qi_{k}$) for each of the 23 essential nutrients as the ratio of the amount of that nutrient contained ($a_{k}$) in 2000 kcal of a country’s average diet to their RDI values [47].

(11) $$qi_{k}=\frac{2000}{E_{k}}\times \frac{a_{k}}{RDI_{k}}$$

Here, 2000 represents the total daily energy needs of an individual (in kcal) and $E_{k}$ is the amount of daily per capita caloric intake (kcal). If the $qi_{k}$ value is > 1, the diet is considered nutrient dense. The NBS was calculated by averaging the $qi_{k}$ of all 23 essential nutrients (Equation (2)):

(12) $$NBS=100\times \left(\frac{\sum_{k=1}^{23}qi_{k}}{23}\right)$$

As outlined by Fern et al. [28], if the $qi_{k}$ for a given nutrient is > 1, it is truncated to 1 based on the rationale that the requirement for a specific qualifying nutrient is already met. If $qi_{k}$ is ≤ 1, it remains unchanged. An NBS of 100 is achieved if a food satisfies 100% of the daily dietary requirement for all 23 qualifying nutrients in a 2000 kcal diet. Conversely, a value of 0 implies that none of the qualifying nutrients are contained in the diet. The RDI values for all essential nutrients are listed in Table S1.

The procedure to calculate the disqualifying nutrient score (DNS) is similar to that for NBS except that instead of essential (qualifying) nutrients, the intake ($a_{j}$) of nutrients of health concern (disqualifying nutrients) is compared against their daily reference values (DRVs; FDA [47]). We first calculate the disqualifying index ($di_{j}$) for each of the four nutrients of health concern (j = 1:4; sugar, total fats, saturated fats and cholesterol):

(13) $$di_{j}=\frac{2000}{E_{j}}\times \frac{a_{j}}{DRV_{j}}$$

Again, 2000 kcal represents the total daily energy needs of an individual and $E_{j}$ is the amount of daily per capita caloric intake (kcal). The $di_{j}$ values of four nutrients were averaged to calculate a single di score. If this average di came > 1, its value was truncated to 1. The DNS was then calculated by subtracting this di from 1 and scaled between 0 and 100 as (see [11] for details):

(14) $$DNS=100-100\times \left(\frac{\sum_{k=1}^{4}di_{k}}{4}\right)$$

A higher DNS value implies the diet is low in disqualifying nutrients and a low value implies the diet is “compromised” because it contains disqualifying nutrients in values higher than the DRV in a 2000 kcal diet. Similar to NBS, as it is normalized by total calories, DNS reflects how dense the diet is in nutrients of health concern.

Our analysis included 23 qualifying nutrients and four disqualifying nutrients because of their well-known beneficial or adverse effects [14,28]. To address the nutritional requirements for Swiss healthy adults, we imported the Dietary Reference Intake (DRI) from the second edition of D-A-CH Reference Levels for Nutrient (edited by nutritionists and dietitians in Germany, Austria and Switzerland) for 21 of total 27 nutrients under analysis. For nutrients missing in this guideline (e.g., cholesterol, total sugar), we referred to Dietary Guidelines for Americans as in previous studies [14,28].

The 82 food items from FAO Food Balance Sheet were matched with the food commodities in Swiss Food Composition Database (http://www.naehrwertdaten.ch/) to estimate the amount of calorie and nutrients they supply. For nutrients like vitamin K, copper, selenium and choline, we imported data from United States Department of Agriculture (USDA) Food Composition Database [48] because Swiss database does not have this data (see Supplementary Table S2 for all qualifying and disqualifying nutrients, as well as their DRIs for a healthy adult). This was done to consider all 23 essential nutrients proposed by the nutrient balance framework of Fern et al. [28] in our calculations rather than skipping a few. We acknowledge that future studies should apply local food composition data for all nutrients if available and assume that the difference in contents of these nutrients between Swiss and US food products do not significantly affect our results.

In order to calculate the third nutrition quality indicator (PAN), we applied the estimated average requirement “cut-point” (EAR-CP) method to estimate the % of people with intakes of a particular nutrient above a demographically-weighted requirement threshold (see Carriquiry [49] and Chaudhary et al. [14] for details). Unlike NBS above, that provides a measure of nutrient adequacy at an individual level, PAN is a population (national) level indicator of nutrient adequacy. We could only include 16 essential nutrients for which EAR values and other data was available for analysis. The PAN indicator was calculated in three steps.

We first constructed a normal distribution of daily per capita intake amounts for each nutrient around the mean by applying a nutrient-specific coefficient of variation (CV) taken from Arsenault et al. [50]. When a CV was not available, it was assumed to be 25% [51]. Next, the age and gender specific EAR of each nutrient [52] was weighted with the population size in each age and gender group [53] to obtain a single weighted EAR (WtdEAR) value. Finally, we applied the EAR-CP method to juxtapose the WtdEAR value with the normal distribution of nutrient intake for each nutrient, to estimate the % of a country’s population with intakes above the WtdEAR threshold. The PAN indicator is reported as the simple mean of these population % across 16 nutrients. See Supplementary Table S2 for values of all parameters used in nutritional quality assessment.

### 2.4. Environmental Impact Analysis

For evaluating environmental impact associated with different dietary scenarios, we linked the consumption amounts of 82 food commodities (gram per capita per day) to commodity-specific footprints regarding GHG emission, freshwater use, cropland use and nitrogen and phosphorus application. We adopted the mean footprint values in the form of environmental impacts per gram of individual foods from Springmann et al. [20].

They imported the GHG emission factors associated with non-carbon dioxide emissions of agriculture from existing studies on crops, livestock, fish and seafood [54,55,56,57,58]. Data on cropland and blue water use (i.e., freshwater from surface and groundwater) were adopted from International Model for Policy Analysis of Agricultural Commodities and Trade (IMPACT) model [59] which accounts for the impact from primary production. Data on nitrogen and phosphorus application to produce different food groups were adopted from International Fertilizer Industry Association [60]. For all impact categories except GHG emissions, environmental footprints of animal products represent feed-related impacts. The ratio of wild-capture and farmed fish as well as seafood products was additionally considered in aquaculture production.

Supplementary Table S3 shows that the greenhouse gas footprint is highest with beef and lamb (~30 gCO_2_eq/g), followed by pork (~3 gCO_2_eq/g), eggs, milk, rice and palm oil (~1–2 gCO_2_eq/g). The freshwater footprint is high for animal-sourced products, sugar, legumes and rice (0.5–1 m^3^/kg) compared with other products. The cropland use is high for legumes, vegetable oils & oil crops, nuts & seeds and animal-sourced products (5–11 m^2^/kg). The nitrogen footprints were high for animal-sourced products, cereals (wheat, rice, maize), oil crops, nuts & seeds and fruits & vegetables (10–50 kgN/kg). The phosphorus footprints mainly followed the trends in nitrogen footprint values.

Please note that the 95% confidence intervals around these footprint values were not available. Also, these values represent global average footprints as Swiss-specific values were not available for use in our analysis. This might under or overestimate the environmental footprint of diets depending upon whether the production system of a particular food item consumed in Switzerland is more intensive or extensive as compared to the global average. We acknowledge that the environmental impacts may vary a lot depending upon the region and production methods [30] and future studies should strive for including footprints for the region from where the food is sourced. For footprints of foods produced within Switzerland, the ecoinvent and the SALCA database can be useful [61].

There are many other studies providing the environmental footprints of individual or food groups mainly using the life cycle assessment (LCA) approach (see review in Reference [30]). However, the reason we used the above food-group specific environmental footprints from Springmann et al. [20] (that are mainly based on the IMPACT model [59]) is because the diet-related environmental impacts calculated using these footprints can be directly compared to the recently calculated food-related planetary boundaries by them [20]. This is because the food group footprints and the respective planetary boundaries calculated by them follow a consistent modelling framework, making it possible to directly compare them. On the other hand, the footprints calculated using the LCA approach are not directly comparable to the food-related planetary boundaries calculated by Springmann et al. [20] because of differences in underlying assumptions, data sources and modelling approaches.

To sort out the issue of how much reduction in environmental footprint is required from current levels or how much increase in footprints is acceptable, the concept of environmental planetary boundaries provides an excellent framework [2,7]. The planetary boundaries if transgressed, increase the risk for harm to the stability of the Earth system and thus can jeopardize human health [7]. The recently published EAT-Lancet commission report on Healthy Diets from Sustainable Food Systems also used these footprints and planetary-boundary values to compare the environmental sustainability of diets under different scenarios [2].

We divided the mean planetary boundaries calculated by Springmann et al. [20] with the global population (year 2011) to come up with the per capita food related planetary boundaries. The calculated planetary boundaries for the greenhouse gas emissions, cropland use, freshwater use, nitrogen application and phosphorus application are: 1867 gCO_2_eq, 5.01 m^2^, 786 L, 27.4 gN and 6.4 gP, respectively (in per capita per day). Finally, we compared the individual environmental footprint under each diet with their per capita food-related planetary boundaries to understand which diets are acceptable from an environmental point of view.

### 2.5. Economic Assessment

We collected the price data of 82 food commodities (in Swiss Francs per kg) considered in this study from the online website of the largest food retailer in Switzerland (*Migros,* www.leshop.ch). The cost of current and all alternative daily average diets were then calculated by multiplying the price of each food item with its daily intake amount (g/day). We also examined the correlation between the health based indicator (reduced DALYs), the three nutrition quality indicators (NBS, DNS, PAN), five environmental footprint indicators as well as the economic cost to understand synergies and trade-offs while shifting from current to alternative diets.

## 3. Results

### 3.1. Health Impact

We found that alternative diets with lower amounts of animal-sourced foods compared to the current Swiss diet, would contribute to a positive health impact, decreasing the risk of adverse outcomes from major chronic diseases. In the year 2016, more than 36,500 Swiss died from the coronary heart disease, stroke, type 2 diabetes and cancers with the health cost in terms of DALYs was 591,095 [6]. As shown in Table 2, the transition to the VGN scenario, according to our health impact model, would have the most health benefits for Swiss population, avoiding around 21,000 disability adjusted life years (DALYs) [95% CI, 16,516–25,460] (Table S4).

The RSN scenario, which follows the recommendation of Swiss Nutrition Society, is the next best diet in terms of human health benefits avoiding more than 15,000 DALYs per year due to chronic diseases in the country. This means that adoption of RSN scenario can lead to a reduction of 2.67% in adverse health outcomes (15,756 ÷ 59,1095 = 0.0267).

The health benefits of transitioning to the other vegetarian scenarios (PST, VGT and FXT) are 25–50% of the above two diets and the estimated health benefit from TAX and HGD scenarios was very small. Due to the similarity between TAX scenario and current eating pattern, the estimated health and environmental benefits are not remarkable. We found that following the meat oriented (MTO) or the protein oriented (PTO) alternative diets with increased consumption of animal resource products would increase the risk of the four disease states considered in this study, associated with an additional 24,000 DALYs approximately (Table 2).

**Figure 1:**
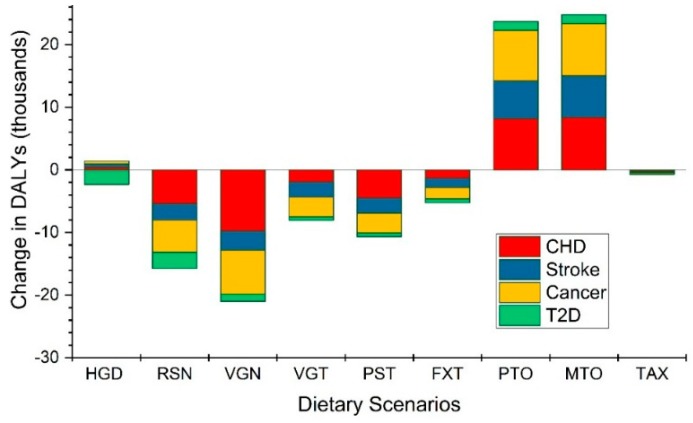
The human health consequences of shifting to an alternative diet. Negative numbers indicate human health benefit and positive numbers indicate an increase in adverse health outcomes. Four disease states used in the health impact model are ischemic or coronary heart disease (CHD), stroke, type 2 diabetes mellitus (T2D) and total cancer. Abbreviations: Healthy Global Diet (HGD), diet following the recommendation of Swiss Society in Nutrition (RSN), Vegan diet (VGN), lacto-ovo Vegetarian diet (VGT), lacto-ovo Pescatarian diet (PST), Flexitarian diet (FXT), protein-oriented diet (PTO), meat-oriented diet (MTO) and food greenhouse gas tax diet (TAX).

Figure 1 shows the disease-specific DALYs change (see Supplementary Table S4 for all numbers including the 95% confidence intervals). We found that the majority of health benefits under the alternative diets occur due to a reduction in risk for coronary heart disease (CHD) followed by cancer and stroke incidents.

For example, under the RSN scenario, almost 34% of the health benefits could be attributed to the reduced risk of coronary heart disease (CHD) while 30% to the reduced risk of cancer. The beneficial impact of dietary change towards RSN on type 2 diabetes (T2D) risk was around 10% (Figure 1).

**Figure 2:**
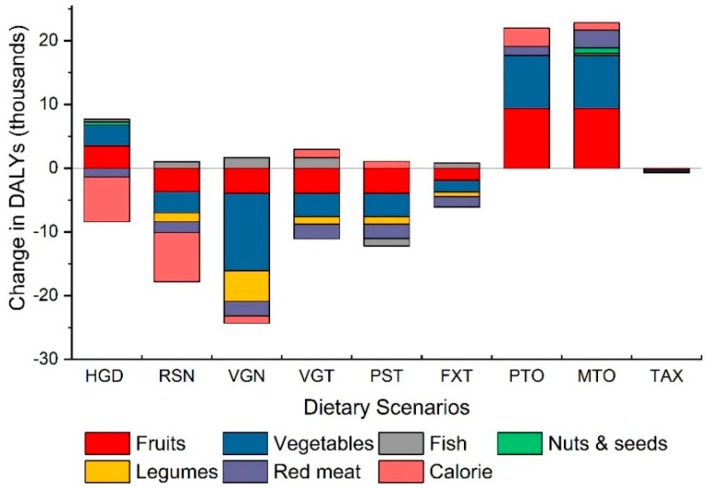
The modelled health impact associated with six dietary factors and the weight related factors (overweight & obesity combined shown as calories) are shown for all nine alternative scenarios. Negative numbers indicate human health benefit and positive numbers indicate an increase in adverse health outcomes.

As shown in Figure 2, when the health benefits under each alternative diet were differentiated according to the six dietary and two weight-related risk factors, we found highest health benefits were due to an increase in vegetable intake over current amounts. The rest of the modelled DALYs reduction were attributable to the increase in intake of fruit and legumes and the elimination of red meat. The potential health benefit from increasing intake of nuts and seeds is projected to be negligible because the average intake already meets the recommended levels in Switzerland. The two weight related risk factors (overweight and obesity) also contributed negligibly to the health benefits under all scenarios except under the RSN (Swiss nutrition recommendations) and HGD (healthy global diet), whose guidelines call for substantial reduction in the total daily calorie intake (~2300 kcal compared with ~3100 kcal in current diet). In these two scenarios, the lower calorie intake (and therefore reduced risk of overweight and obesity related disease burden) is projected to be the most significant factor contributing positively to the health. On the other hand, the adverse consequences (mainly increase in CHD, stroke and cancer) when shifting to PTO and MTO diets are mainly due to reduced fruits & vegetable intake compared with current levels.

### 3.2. Nutrition Quality

We found that the RSN scenario will improve the nutrient balance score (NBS, Equation (12)) of Swiss diets by >5 (from current 93 to 99) while keeping the disqualifying nutrient score (DNS, Equation (14)) and the % population with adequate nutrition (PAN) score at almost the same levels as currently (Table 2). In terms of individual nutrients, the adoption of RSN scenario will lead to meeting the daily recommended levels of 23 out of 27 nutrients considered in this study (marked as green in Table 3).

Compared to the RSN scenario, the current Swiss diet is comprised of almost four times more meat (129 g currently vs. 33 g recommendation), three times higher vegetable oil, two times higher fish products and 1.5 times higher cereals (e.g., wheat, maize, rice) and roots. On the other hand, the intake of legumes, nuts & seeds is much lower than the recommended levels. The intake of fruits, vegetables, dairy and eggs is closer to the recommended levels (Table 1).

All other eight alternative dietary scenarios considered here will lead to deterioration in one or the other indicator of nutrition quality (Table 2 and Table 3). For example, as shown in Table 3, while the current Swiss diets meets the daily requirement of choline and vitamin B_12_, the adoption of the VGN scenario will lead to low intakes of these two essential nutrients because they are primarily supplied by the animal based products which are absent from VGN diet and the addition of fruits, vegetables & legumes do not compensate for these nutrients.

We found that the PAN score reduces by almost 10% under the VGN and HGD scenarios, meaning that just 87% of Swiss population will be achieving their daily essential nutrient intake goals as compared to 96% under current diet. The protein (PTO) and meat oriented (MTO) dietary scenario lead to reduction in DNS because of increased intake of disqualifying (harmful) nutrients such as cholesterol and total fats (Table 3). The nutrition quality indicator scores changed negligible under the TAX scenario.

### 3.3. Environmental Impact

**Figure 3:**
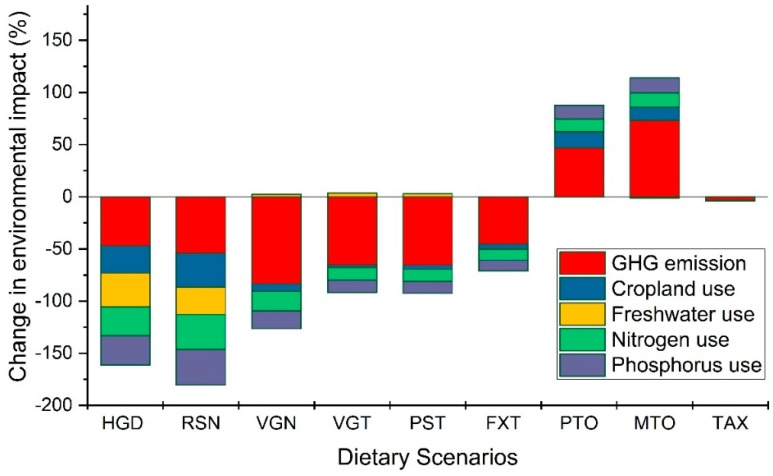
The environmental footprint of the nine alternative diets relative to the current diet. It can be seen that shifting from current to the meat oriented (MTO) and protein oriented (PTO) diets will increase the environmental footprint across all five domains (GHG, water, land, nitrogen and phosphorus) while a shift to the RSN diet (recommended by Swiss society of nutrition) will lead to maximum reduction in footprints. See Supplementary Table S5 for contribution of different food groups to the total environmental footprint.

With data from IMPACT model [59] and commodity-specific environmental footprints reported in previous studies [20], we found that shifting to a diet recommended by Swiss society of nutrition (RSN) will be the most beneficial for the environment, cutting down the daily food related environment footprint by ~36% on average across five domains considered here. For example, the current greenhouse gas, water, land, nitrogen and phosphorus footprint of Swiss diet (in per capita per day) are: 2268 gCO_2_eq, 590 L, 4.38 m^2^, 29.03 gN and 5.23 gP, respectively (Table 4). Results show that adoption of RSN diet will reduce these footprints by 54%, 26%, 32%, 33% and 34%, respectively (Figure 3; Table 4).

The changes in individual environmental footprint varied with the type of scenario. For example, while the vegan (VGN), vegetarian (VGT) and Pescatarian scenario leads to 83%, 63% and 64% reduction in GHG emissions from current levels, the freshwater use stays almost the same (slight increase of ~3% in each of these scenarios, reflecting potential trade-offs between different environmental objectives).

Table 4 shows the calculated environmental footprints associated with daily per capita diets and their comparison with the food-related planetary boundaries. We found that the current average per capita Swiss diet transgresses the greenhouse gas emission and nitrogen use planetary boundaries (shown in red in Table 4) and barely meets the other planetary boundaries (shown in green in Table 4). A shift to RSN, vegetarian and flexitarian diets will bring all five footprints well under the planetary boundaries. On the other hand, adoption of meat oriented (MTO) and protein oriented diet (PTO) leads to an increase in greenhouse gas footprint by ~50%, and all other footprints except freshwater footprint by ~20% (Figure 3) and transgression of almost all five planetary boundaries.

### 3.4. Economic Impact

**Figure 4:**
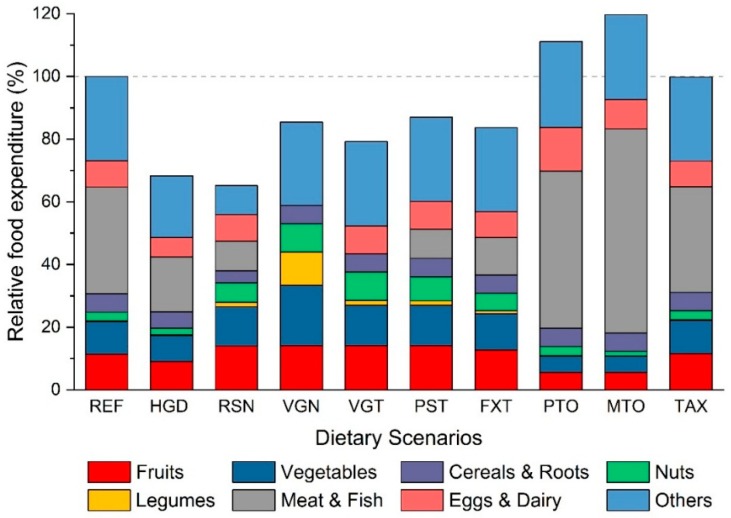
Cost of nine alternative diets relative to the current diet (REF = 100%). It can be seen that shifting from current to the meat oriented (MTO) and protein oriented (PTO) diets will increase the cost by 10–20% while adopting a diet based on global (HGD) or Swiss nutrition guidelines (RSN) will decrease the cost by ~35%. See Supplementary Table S6 for cost due to intake of individual food group.

Among the nine alternative diets in our analysis, the scenario following Swiss dietary guideline (RSN) is the cheapest in terms of economic cost based on the food product average retail prices in the largest Swiss supermarket (MIGROS). A shift to RSN scenario is projected to save more than one third of the daily food expenditure primarily because of replacement of expensive meat products with relatively cheaper fruits, vegetables, nuts and legumes (Figure 4). With larger amounts of animal products especially meat in PTO and MTO scenarios, the cost increases by 10–20% from current levels.

### 3.5. Correlation Analysis

Table 5 shows the Spearman rank correlation between 11 indicators of sustainability employed here. We found a statistically significant positive correlation between health benefits and the average nutrition density (Avg. ND) score (i.e., mean of NBS and DNS indicators) with a Spearman correlation equal to ~0.70. This is interesting because the correlation between the health benefit indicator (reduced DALYs) and the density of essential nutrients (NBS) or the density of harmful nutrients (DNS) is not significant (0.10 and 0.37, respectively).

The correlation between health & nutrition indicators and environmental footprints was negative and significant (ρ > 0.75) except for water footprint for which there was no statistically significant correlation. The five environmental footprints were positively correlated with each other except for greenhouse gas and the water footprint which were negatively correlated.

Table 5 shows that the health and nutrition indicators were negatively correlated with the cost of diet except for PAN indicator meaning that a more nutritious diet has lesser cost. All environmental indicators were positively correlated with the cost (although this correlation was not significant for water footprint) meaning that a diet with a higher environmental footprint is also costlier for consumers. Though limited by a small sample size (e.g., *N* = 9), our preliminarily results are encouraging and show that the transition to a healthy and more sustainable diet in general would not increase economic pressure on consumers.

## 4. Discussion

Our multi-indicator quantitative comparison of current and nine alternative diets for the Swiss population revealed that transitioning towards a healthy diet following the guidelines of Swiss society of nutrition will bring the highest sustainability benefits as it is projected to reduce 36% of the environmental footprint, save one third of expenditure on food and lower the adverse health outcome by 2.67% compared to the current diet (Table 2). Achieving this sustainable diet would entail a high reduction in the intake of meat and vegetable oils, a moderate reduction in cereals, roots and fish products and at the same time increased intake of legumes, nuts, seeds, fruits and vegetables (Table 1).

On the other extreme, our modelling results show that transitioning towards a meat or protein oriented diet is the least sustainable option out of all 10 diets considered here as it is projected to result in large increases in diet related adverse health outcomes, environmental footprint, daily food expenditure and low intakes in essential nutrient with risk of leading to deficiency (for vitamin C, fibre, potassium and calcium; Table 2 and Table 3).

Overall, our results are in line with other recent studies suggesting that shifting to a healthy diet is not only good for human health but also for the environment [2,8,18,24,29] and that replacing meat with plant-based alternatives such as legumes, improves the nutrition quality and reduces the environmental footprints [14]. This is also confirmed by our correlation analysis (Table 5) where we found a significant negative correlation between health benefits and environmental footprints suggesting that healthier and nutrient dense diet can be in line with the goal of environmental sustainability. In addition, results show that daily food expenditure is positively correlated with environmental footprints and negatively correlated with health and nutrient density indicators. In other words, a healthy & nutritious diet that has low impact on the environment need not be expensive. It can be explained by the high meat price in Switzerland and shifting from current to nutritious diet (e.g., RSN scenario) entails cutting down on meat intake, ultimately leading to economic savings as the meat replacement products such as legumes and nuts are cheaper than meat. This is encouraging evidence showing a synergy between environmental, health and nutritional sustainability.

Our analysis presents several other insights useful from a sustainability point of view. For example, we found that while adopting a vegetarian or vegan diet has many benefits such as reduced adverse health outcomes, food expenditure and greenhouse gas footprint (Table 2), it might lead decreased intake of essential micronutrients (e.g., Vitamin B_12_, Choline and Calcium; Table 3). Such insights were only possible because we employed a total of 10 indicators of dietary sustainability over four dimensions (human health, nutrition, environment and economics). A multi-indicator analysis enabled us to figure out possible trade-offs not only across the four dimensions of sustainability considered here but also between the different indicators of the same dimension (e.g., GHG vs. water footprint for vegetarian diet).

In our comprehensive nutrition quality analysis, we compared the intake of 23 essential and 4 harmful nutrients with their daily recommended levels and found that even a Swiss diet meeting the essential nutrients and health recommendations (RSN; HGD) exceeds in the intake of harmful (disqualifying) nutrients such as fats, cholesterol and sugar (Table 3). Therefore, the disqualifying nutrient score (DNS), is currently low at <10 for all scenarios meaning that foods high in these nutrients of health concern needs to be cut down. We also found that the daily recommended levels of four essential nutrients (calcium, potassium, fibre and choline) are barely met in the current Swiss diet (ratio ~ 1) and therefore, food containing these nutrients should be encouraged in daily diet (Table 3).

In our environmental analysis, we found that the current greenhouse gas emission and nitrogen footprint of daily average Swiss diet exceeds the respective food related planetary boundaries, meaning that food items high in GHG emissions (e.g., beef, lamb, pork; Supplementary Table S3) and requiring high fertilizer application (e.g., oil seeds, rice or grain-fed poultry, pork) should be discouraged in order to meet environmental sustainability goals.

Our analysis comes with several limitations and uncertainties that should be considered while interpreting the results. Below, we list these limitations and sources of uncertainties as well as research gaps that needs to be filled through future efforts.

First, we relied on the current Swiss diet data from the UN FAO’s database [33] which does not provide any uncertainty around its estimates, might suffer from errors and only provides the food intake aggregated across 82 broad food groups for Switzerland. In practice, the Swiss population eats thousands of individual food items (processed, cooked and modified from raw agricultural commodities to a varying degree) differing widely in their environmental footprint and nutrition content. For example, our study predicts remarkable low intake of vitamin B_12_ in the vegan diet scenario compared with current levels. However, people can obtain vitamin B_12_ from plant foods such as edible algae [62] or dietary supplements, which are not included in 82 food categories considered here. A more detailed dietary database along with the mean and standard deviation estimates of food consumption is needed to overcome the potential errors in the calculated results.

Second, the Swiss food composition database (http://www.naehrwertdaten.ch/ or USDA [48]) and the environmental emission factor database [20] that we use, do not provide the uncertainty intervals around their estimates and thus, we could not propagate this to the end result on nutrition quality and environmental indicators. For the nutrition, we did not take into account the bioavailability or the interaction of nutrients with one another that might affect the actual absorption of nutrients into the body [63].

For the environmental emission factors, we used the global average values that were publicly available but future studies should use the country-specific emission factor values. Also, we could not consider the impacts of dietary change on other environmental categories such as biodiversity loss, ecosystem services [64,65,66,67,68,69] or human toxicity through air/water pollution due to a lack of emission factors for all food items considered here.

Third, our health impact estimates (DALY values, Table 2) are likely an underestimate because we only included the health impacts due to intake of six food groups (red meat, fruits, vegetables, legumes, fish, nuts & seeds) and were not able to model the health consequences of changes in consumption of other food groups or components such as dairy, vegetable oils, whole grains, dietary fibre and so forth. [21,70,71,72,73,74,75]. We simply used the relative risk factors (RR) for these food groups because the epidemiological evidence on impact of these foods on human health is robust and statistically significant and has been employed recently in the global scale EAT-Lancet commission calculating dietary impacts on health [2]. More research is required to come up with reliable data on risk of other food groups to human health and address other limitations of epidemiological research [76].

The underestimation on health impact can also be attributed to the limited number of diseases considered in our model (CHD, T2D, cancer, stroke, obesity, overweight) which simply follows the guidelines of Willet at al. [2] although it might be that the six food groups considered here contribute to increased risk of some other diseases as well (e.g., Alzheimer, dementia etc.). In the health impact assessment, we were able to propagate the uncertainty in relative risk parameters to the calculated indicators (Supplementary Table S2).

Fourth, we used the average price of 82 food groups from the largest food retailer website in our cost analysis (Figure 4) but the cost of food items may differ depending upon its origin or whether it is processed and several other factors. Once more detailed food intake data are available, future studies should match them with their respective prices.

Finally, we only included nine popular alternative dietary scenarios but one can also obtain a sustainable diet using an optimization algorithm that starting with the current diet, generates optimum intake amounts of different food items meeting multiple nutritional, environmental or other constraints (see e.g., Jalava et al. [19]).

## 5. Conclusions

Overall, we estimated the impact of dietary shifts on environment, human health, nutrition quality and economic dimensions with multiple indicators and provide the magnitude of possible benefits for Switzerland if it decides to transition towards sustainable dietary patterns. Importantly, we found synergies among these four dimensions (Table 5) meaning achieving healthy diet need not be expensive and bad for the environment. The health analysis revealed the need for dietary intervention and food policies to fill the Swiss dietary gaps (e.g., overconsumption of meat, vegetable oils and low intake of legumes, nuts & seeds; Table 1). Our nutrition analysis could be a useful basis for designing national level strategies such as increasing the production, availability and affordability of foods high in calcium, potassium and choline whose current intake barely meets the recommended intake levels (Table 3). The environmental analysis showed that the current average diet has higher than acceptable greenhouse gas emissions and nitrogen footprints, threatening to transgress their global planetary boundaries (Table 4) and therefore points towards need for policies that can reduce the embodied environmental impacts in the food items or reduce the consumption of items with high footprints per kg (Supplementary Table S3). We hope our quantitative results will help Swiss policy-makers identify key areas of food system improvement. Our work underscores the need to consider multiple indicators in food sustainability assessment and provides a template to conduct such studies in other countries and settings. To fill the identified data gaps and overcome the challenges in shifting towards sustainable food production, consumption and distribution, it is clear that stakeholders including consumers, producers, researchers and policy-makers, would have to coordinate at national and international level.

## Figures and Tables

**Table 1:** Food consumption (g capita^−1^ day^−1^) under different Swiss dietary scenarios.

Food Items/Scenarios	REF	HGD	RSN	VGN	VGT	PST	FXT	PTO	MTO	TAX
Fruits	265	211	325	330	330	330	295	130	130	269
Vegetables	239	190	291	445	296	296	268	119	119	243
Legumes	5	5	26	84	23	23	16	5	0	5
Nuts and seeds	24	18	50	73	73	62	46	24	13	24
Cereals	192	200	124	192	192	192	192	192	192	189
Meat products	129	63	33	0	0	0	41	201	268	127
Fish & seafood	15	11	6	0	0	27	8	15	15	15
Eggs	25	19	18	0	31	31	23	65	39	25
Dairy products	307	225	330	0	307	307	307	433	307	295
Vegetable oils	71	52	26	71	71	71	71	71	71	71
Roots & tubers	230	150	149	230	230	230	230	230	230	233
Others	298	223	109	295	299	299	298	304	300	298

The scenarios are as follows: current Swiss diet (REF), Healthy Global Diet (HGD), diet following the recommendation of Swiss Society in Nutrition (RSN), Vegan diet (VGN), lacto-ovo Vegetarian diet (VGT), lacto-ovo Pescatarian diet (PST), Flexitarian diet (FXT), protein-oriented diet (PTO), meat-oriented diet (MTO) and food greenhouse gas tax diet (TAX).

**Table 2:** Human health, nutritional, environmental and economic indicator scores under different Swiss dietary scenarios.

Scenario	Human Health	Nutritional			Environmental					Economic
	Reduced DALYs *	NBS	DNS	PAN	GHG	WFP	LFP	NFP	PFP	Cost
REF	-	93.82	0.00	96	2.27	0.59	4.38	29.0	5.23	10.58
HGD	953	95.93	1.35	87	1.20	0.40	3.24	21.0	3.75	7.23
RSN	15,756	98.77	0.00	91	1.04	0.44	2.96	19.3	3.45	6.89
VGN	20,986	88.08	15.41	87	0.38	0.60	4.08	23.6	4.33	9.04
VGT	8049	91.37	0.00	94	0.78	0.61	4.27	25.7	4.61	8.38
PST	10,679	92.68	0.00	95	0.78	0.61	4.21	25.7	4.62	9.21
FXT	5259	93.09	0.00	94	1.24	0.59	4.16	26.0	4.69	8.85
PTO	−23,699	88.76	0.00	95	3.33	0.60	5.04	32.5	5.93	11.76
MTO	−24,788	88.56	0.00	92	3.92	0.58	4.94	33.0	5.98	12.67
TAX	706	93.82	0.00	96	2.20	0.59	4.37	29.0	5.22	10.56

The scenarios are as follows: current Swiss diet (REF), Healthy Global Diet (HGD), diet following the recommendation of Swiss Society in Nutrition (RSN), Vegan diet (VGN), lacto-ovo Vegetarian diet (VGT), lacto-ovo Pescatarian diet (PST), Flexitarian diet (FXT), protein-oriented diet (PTO), meat-oriented diet (MTO) and food greenhouse gas tax diet (TAX). For each of the 10 indicators, the best and worst scores are marked in green and red, respectively.* See Supplementary Tables S4–S6 for the food group specific results and 95% confidence intervals. * A negative value under health indicator (i.e., change in DALYs per year) means the diet is bad for human health relative to the current diet. The nutrition quality indicators are: nutrient balance score (NBS), disqualifying nutrient score (DNS) and % population share with adequate nutrients (PAN). The nutrition indicators vary from 0–100 with a higher score signifying a nutritious diet meeting recommended levels. Five food related environmental footprints (per capita per day) are: greenhouse gas (GHG in kg CO_2_eq), water (WFP in m^3^), land (LFP in m^2^), nitrogen (NFP in gN) and phosphorus (PFP in gP). Cost is the daily expenditure on food in Swiss Francs (CHF).

**Table 3:** The ratio of daily nutrient intake amounts and their daily recommended levels under the current Swiss diet (REF) and nine alternative dietary scenarios.

Nutrients/Scenarios	REF	HGD	RSN	VGN	VGT	PST	FXT	PTO	MTO	TAX
**Qualifying nutrients**										
Folate	1.3	1.5	1.6	1.6	1.5	1.5	1.4	1.0	1.0	1.3
Niacin	1.6	1.6	1.6	1.6	1.4	1.5	1.6	1.6	1.6	1.6
Pantothenic acid	1.6	1.6	1.6	1.4	1.4	1.4	1.5	1.6	1.6	1.5
Vitamin B_2_	1.6	1.6	1.6	1.1	1.3	1.4	1.4	1.6	1.6	1.5
Thiamin	1.6	1.6	1.6	1.6	1.6	1.6	1.6	1.6	1.6	1.6
Vitamin A	1.6	1.6	1.6	1.6	1.6	1.6	1.6	1.6	1.6	1.6
Vitamin B_12_	1.6	1.6	1.6	0.1	0.8	1.1	1.3	1.6	1.6	1.6
Vitamin B_6_	1.6	1.6	1.6	1.6	1.6	1.6	1.6	1.6	1.6	1.6
Vitamin C	1.2	1.4	1.6	1.6	1.5	1.5	1.4	0.7	0.7	1.3
Vitamin E	1.6	1.6	1.6	1.6	1.6	1.6	1.6	1.6	1.6	1.6
Vitamin K	1.6	1.6	1.6	1.6	1.6	1.6	1.6	1.0	1.0	1.6
Calcium	1.0	1.0	1.4	0.7	1.0	1.0	1.0	1.0	0.9	1.0
Copper	1.6	1.6	1.6	1.6	1.6	1.6	1.6	1.6	1.6	1.6
Iron	1.5	1.6	1.6	1.6	1.6	1.6	1.6	1.4	1.5	1.6
Magnesium	1.6	1.6	1.6	1.6	1.6	1.6	1.6	1.4	1.4	1.6
Phosphorus	1.6	1.6	1.6	1.6	1.6	1.6	1.6	1.6	1.6	1.6
Potassium	1.1	1.2	1.5	1.4	1.2	1.2	1.2	0.9	1.0	1.1
Selenium	1.6	1.6	1.6	1.6	1.6	1.6	1.6	1.6	1.6	1.6
Zinc	1.6	1.6	1.6	1.6	1.6	1.6	1.6	1.6	1.6	1.6
Polyunsaturated fats	1.6	1.6	1.6	1.6	1.6	1.6	1.6	1.6	1.6	1.6
Choline	1.2	1.2	1.3	0.6	1.0	1.1	1.1	1.6	1.5	1.2
Dietary fibre	1.3	1.6	1.6	1.6	1.6	1.5	1.5	0.9	1.0	1.3
Protein	1.6	1.6	1.6	1.4	1.3	1.4	1.5	1.6	1.6	1.6
**Disqualifying nutrients**										
Total fats	1.4	1.4	1.4	1.4	1.5	1.4	1.4	1.5	1.4	1.4
Saturated fats	2.2	2.2	2.2	1.6	2.1	2.1	2.2	2.4	2.3	2.2
Cholesterol	1.3	1.1	1.1	0.2	0.9	1.0	1.0	2.0	1.8	1.2
Total sugars	2.0	1.5	2.3	2.2	2.0	2.0	2.0	1.6	1.6	2.0

The nutrient intakes meeting the recommended levels are shown in green while those not meeting their recommended levels are shown in red.

**Table 4:** The daily per capita food related environmental footprint of current Swiss diet (REF) and nine alternative diets.

Dietary Scenario	GHG Emission (gCO_2_eq)	Cropland Use (m^2^)	Freshwater Use (litres)	Nitrogen Use (gN)	Phosphorus Use (gP)
**REF**	2267	4.38	590	29.03	5.23
**HGD**	1202	3.24	400	21.00	3.75
**RSN**	1036	2.96	436	19.31	3.45
**VGN**	377	4.08	604	23.56	4.33
**VGT**	783	4.27	611	25.67	4.61
**PST**	779	4.21	608	25.70	4.62
**FXT**	1238	4.16	590	26.03	4.69
**PTO**	3326	5.04	596	32.46	5.93
**MTO**	3923	4.94	583	33.00	5.98
**TAX**	2196	4.37	591	28.99	5.22

The footprints meeting their planetary boundaries are shown in green while those not meeting are shown in red. The daily per capita food related planetary boundaries for the greenhouse gas emissions, cropland use, freshwater use, nitrogen application and phosphorus application are: 1867 gCO_2_eq, 5.01 m^2^, 786 L, 27.4 gN and 6.4 gP, respectively (Springmann et al. [20]).

**Table 5:** Spearman rank correlation coefficient (ρ) between different indicators of dietary sustainability used in this study.

	DALYs	Average ND	NBS	DNS	PAN	GHG	WFP	LFP	NFP	PFP	Cost
DALYs	-										
Average ND	**0.70**	-									
NBS	0.10	0.40	-								
DNS	0.37	**0.64**	−0.18	-							
PAN	−0.38	**−0.51**	0.03	**−0.73**	-						
GHG	**−0.93**	**−0.58**	0.03	−0.46	0.35	-					
WFP	0.27	−0.31	**−0.54**	−0.20	0.49	−0.43	-				
LFP	**−0.73**	**−0.83**	**−0.53**	**−0.50**	**0.68**	**0.61**	0.41	-			
NFP	**−0.79**	**−0.79**	**−0.49**	**−0.50**	**0.61**	**0.75**	0.21	**0.93**	-		
PFP	**−0.78**	**−0.78**	**−0.48**	**−0.50**	**0.62**	**0.74**	0.21	**0.92**	**0.996**	-	
Cost	**−0.63**	**−0.63**	**−0.63**	−0.27	0.52	**0.58**	0.28	**0.87**	**0.90**	**0.92**	-

Values in bold print indicate statistically significant (*P-value* < 0.1). Average ND is the average of NBS and DNS.

## Supplementary Materials

The following are available online at https://www.mdpi.com/2072-6643/11/4/856/s1, Table S1: Raw data and parameter values used in the health impact assessment model. Table S2: Raw data and parameter values used in the nutrition quality assessment. Table S3: Characterization factors for different food groups giving environmental footprint per kg of a food item used for environmental assessment. Table S4: Detailed results on health impact assessment including 95% value for indicators. Table S5: Contribution of each food group to the total environmental footprint of daily average per capita Swiss diet. Table S6: Contribution of different food group to the total expenditure on daily average per capita Swiss diet.
